# An optimized approach and inflation media for obtaining complimentary mass spectrometry-based omics data from human lung tissue

**DOI:** 10.3389/fmolb.2022.1022775

**Published:** 2022-11-16

**Authors:** Jessica K. Lukowski, Heather Olson, Marija Velickovic, Juan Wang, Jennifer E. Kyle, Young-Mo Kim, Sarah M. Williams, Ying Zhu, Heidi L. Huyck, Matthew D. McGraw, Cory Poole, Lisa Rogers, Ravi Misra, Theodore Alexandrov, Charles Ansong, Gloria S. Pryhuber, Geremy Clair, Joshua N. Adkins, James P. Carson, Christopher R. Anderton

**Affiliations:** ^1^ Pacific Northwest National Laboratory (PNNL), Richland, WA, United States; ^2^ Department of Pediatrics, University of Rochester Medical Center, Rochester, NY, United States; ^3^ Structural and Computational Biology Unit, European Molecular Biology Laboratory, Heidelberg, Germany; ^4^ Texas Advanced Computing Center (TACC), University of Texas at Austin, Austin, TX, United States

**Keywords:** MALDI, mass spectrometry imaging, laser capture microdissection, metabolites, lipids, proteins, molecular pulmonology

## Abstract

Human disease states are biomolecularly multifaceted and can span across phenotypic states, therefore it is important to understand diseases on all levels, across cell types, and within and across microanatomical tissue compartments. To obtain an accurate and representative view of the molecular landscape within human lungs, this fragile tissue must be inflated and embedded to maintain spatial fidelity of the location of molecules and minimize molecular degradation for molecular imaging experiments. Here, we evaluated agarose inflation and carboxymethyl cellulose embedding media and determined effective tissue preparation protocols for performing bulk and spatial mass spectrometry-based omics measurements. Mass spectrometry imaging methods were optimized to boost the number of annotatable molecules in agarose inflated lung samples. This optimized protocol permitted the observation of unique lipid distributions within several airway regions in the lung tissue block. Laser capture microdissection of these airway regions followed by high-resolution proteomic analysis allowed us to begin linking the lipidome with the proteome in a spatially resolved manner, where we observed proteins with high abundance specifically localized to the airway regions. We also compared our mass spectrometry results to lung tissue samples preserved using two other inflation/embedding media, but we identified several pitfalls with the sample preparation steps using this preservation method. Overall, we demonstrated the versatility of the inflation method, and we can start to reveal how the metabolome, lipidome, and proteome are connected spatially in human lungs and across disease states through a variety of different experiments.

## Introduction

Disease states are biomolecularly multifaceted and span multiple phenotypic cell states, therefore it is important to understand diseases on all levels, across cell types, and within microanatomical tissue compartments. The lung is comprised of a variety of cell types organized in many distinct spatially defined functional tissue units. As such, to fully understand pulmonary health and disease it is essential to have means to assess spatial heterogeneity at the molecular level. In addition, a variety of pulmonary diseases affect the pulmonary tissue heterogeneously, such as bronchopulmonary dysplasia (BPD) (Kalikkot Thekkeveedu et al., 2017), idiopathic pulmonary fibrosis (IPF) (Wolters et al., 2014; Neumark et al., 2020), and chronic obstructive pulmonary disease (COPD) (Vogt et al., 2012). Mass spectrometry (MS) mediated approaches offer in-depth detection of a wide range of molecules directly from tissue samples (Chughtai and Heeren, 2010; Taylor et al., 2021). Each MS approach can then be methodologically optimized to specifically determine the abundances of different classes of biomolecules. The use of multiple MS-based assays can be employed for analyzing the same sample to reveal interdependent information about the biomolecular state of a sample. Notably, metabolomics and lipidomics can provide unique insights into the function of genes and proteins, as metabolites and lipids are directly linked to cellular function, response to injury, and progression to disease (Fiehn, 2002; Patti, G., Yanes, O., Siuzdak, 2012; Samarah et al., 2020; El-Achkar et al., 2021; Hansen et al., 2021).

Integrative multi-omics analyses can empower more effective investigations and a higher understanding of complex biological systems. The metabolite, protein, and lipid extraction (MPLEx) method (Nakayasu et al., 2016; Burnum-Johnson et al., 2017) is a robust analyte extraction method, which enable comprehensive bulk-based omics measurements of said molecular classes (e.g., by using mass spectrometry; MS). MPLEx and MS analysis has been employed on a wide variety of sample types (Nakayasu et al., 2016; Burnum-Johnson et al., 2017; Nicora et al., 2018), including for comprehensive profiling the biomolecules from pulmonary tissues (Dautel et al., 2017; Kyle et al., 2018; Moghieb et al., 2018; Sun et al., 2022). This method, by enabling the extraction of different classes of biomolecules from the same sample, has provided a more holistic view of active biological pathways within samples of interest (e.g., lung). A complementary approach to these bulk-based omics methods is matrix-assisted laser desorption/ionization-mass spectrometry imaging (MALDI-MSI), which is growing in popularity (Palmer et al., 2016), and can provide spatially-resolved omics data from a sample (e.g., ‘a molecular map’) (Spraggins et al., 2016). MALDI-MSI can enable spatially-resolved detection of hundreds of molecular species (small molecules, lipids, peptides, proteins, etc.) simultaneously directly from single cells, tissues, organs, or whole organisms *in situ* (Castellino et al., 2011; Taylor et al., 2021). MALDI-MSI is a soft ionization technique that uses a small organic acid, which is applied to a thin tissue section, to help aid in desorption and ionization of endogenous molecules. In this technique, a laser is used to probe the sample in a serial fashion (i.e., spot-by-spot), where the laser causes desorption/ionization of endogenous molecules at each location that can be measured by a mass analyzer. From this the spatial distributions of the analytes detected can be visualized within a sample. In combination with histochemical (auto)fluorescence, and other mass spectrometry imaging methods, MALDI-MSI can reveal metabolic pathways that are related to disease states and characteristics of metabolic reprogramming across microanatomical tissue regions and cell types (Rocha et al., 2017; Neumann et al., 2019; Nishidate et al., 2019; Blutke et al., 2020).

There are a number of examples of researchers using multimodal and multi-omic approaches for in-depth characterization of pulmonary processes. Van Nuffel et al. recently described their multimodal MSI approach to identify markers of pulmonary arterial hypertension in lung tissue (van Nuffel et al., 2020). Also utilizing a multi-omics approach, Berghmans et al. studied the tumor microenvironment in the pulmonary tissue of patients with advanced non-small-cell lung cancer (Berghmans et al., 2019). MALDI-MSI experiments enabled them to molecularly profile different regions of cancerous lung tissues. Additionally, they linked their MALDI-MSI data to accurate molecule identifications by performing bulk omics measurements using liquid chromatography-mass spectrometry (LC-MS). This allowed for further biological interpretation to be made regarding the molecular profile of advanced non-small-cell lung cancer (Berghmans et al., 2019). Finally, Robinson and coworkers provided another multimodal omics example, where they characterized drug and excipient distributions in rat lung by MALDI-MSI and time-of-flight secondary ion mass spectrometry (Robinson et al., 2021). By employing these two complementary analyses, they began to understand how to best implement the controlled release of microparticle formulations for respiratory illnesses. This work led them to develop more targeted therapeutic strategies, reducing potential side effects (Robinson et al., 2021). These three examples illustrate the power of multimodal MS and multi-omics measurements for understanding biochemical processes within the lung. However, these studies did not explore an optimized sample embedding and handling method for multi-omics measurements, which would enable characterizing and spatially localizing the full breadth of biomolecules in the lung.

Sample preparation requires special considerations when performing multi-omics and spatial omics on the same tissue sample. Particularly, sample preparation can play a large role in the attainable information output of all tissue types, and it is particularly challenging in the lung that can lose micro-structural relationships if deflated. In the case of a MSI experiments, the spatial fidelity of molecules within the tissue can be preserved if the tissue architecture of the airspaces is maintained by inflation with a support matrix prior to being embedded for sectioning. Other considerations are also necessary for preparation for MALDI-MSI experiments, where many inflation and embedding methods are not compatible with this type of analysis (e.g., optimal cutting temperature medium or “OCT” is not compatible with MSI) (Angel et al., 2012; Truong et al., 2021). Additionally, thawing of the sample during inflation could alter the molecular state of endogenous species (i.e., molecular degradation), which would ultimately lead to incorrect biological interpretations and false biomarkers. Furthermore, the inflation medium should not leak polymers that have the potential to alter the mass spectrometric signal or contaminate the instrument. Over the past decade, several inflation medias have been reported to improve the sample preparation of lung tissue for MSI techniques (Zaima et al., 2010; Goodwin, 2012). These inflation strategies include carboxymethyl cellulose (CMC) (Jones et al., 2017), gelatin (Scott et al., 2019), and agarose (Braber et al., 2010). These media can then be used as an embedding method to further stabilize the fragile lung tissue for analysis. More recently, hydroxypropyl-methyl cellulose polyvinylpyrrolidone (HPMC-PVP) has shown promise in being an ideal hydrogel to be used for embedding a wide range of samples, but it has not been fully investigated as a possible media to use for lung inflation (Dannhorn et al., 2020).

Here, we sought to explore and optimize the inflation media used on human lung tissue to maximize the informational output we obtained from bulk and spatial proteomics, lipidomics, metabolomics through a multi-omics approach. By utilizing several different omics techniques, we can begin to spatially map the distribution of broad classes of molecules throughout the lung and verify their identities through bulk omics. Specifically in these experiments, we focused on the use of agarose inflation, as this method has been shown to be a compatible method for micro-computed tomography (microCT) imaging (Hsu et al., 2016). Obtaining the three-dimensional anatomical structure would provide not only accurate registration of molecular data, but also anatomical context of the molecular information obtained, and therefore maximize the use of rare human lung samples in the future. Finally, we performed initial complementary multi-omic MS measurements on lung tissue samples preserved using CMC and HPMC-PVP for comparison to agarose inflation.

## Materials and methods

### Lung inflation and embedding

Lung samples were requested through the BioRepository for Investigation of Diseases of the Lung (BRINDL) (Ardini-Poleske et al., 2017). Prior to preparation the research-consented, donor *en bloc* lungs are maintained on wet ice in transplant buffer (UW or HTK). The lung surfaces were kept moist throughout the inflation process. One lobe was chosen for matrix inflation. After canulation of the main bronchus, stabilized by a zip-tie to reduce leaks, the lobe was warmed by flotation for 5 min in Hanks’ balanced salt solution (HBSS, Corning; Cat. No. 21–022-CV) at 37°C. Agarose, 2% w/vol (low-gelling temperature agarose, Sigma; Cat. No. A9414), freshly made in phenol-free Dulbecco’s Modified Eagle’s Medium (DMEM) media (Gibco; Cat. No. 21063–029) and warmed in a 37°C water bath, was then instilled *via* the bronchial catheter using a 20 ml syringe refilled as needed to inflate the entire lobe. Gentle pressure was applied to the syringe to inject the agarose solution into the lung. Two people were necessary for this process, as one held the lobe and catheter while the other instilled the agarose. Once the lobe was fully inflated the zip-tie was made snug on the bronchus as the catheter was removed. The lobe was then submerged in ice cold HBSS on wet ice for 45 min to solidify the agarose solution. Photographs of the inflated lobe were taken. Tissue blocks, approximately 1 cm × 1 cm x 0.5 cm, were made from the solid lobe by standard protocol on a moist cool surface (Pryhuber and Huyck, 2020). Most tissue blocks were then embedded in a cryomold with 5% CMC (Sigma; Cat. No. 419272, average molecular weight ∼9 kDa) and frozen on a thin aluminum plate over a dry-ice ethanol bath. Blocks were foil-wrapped and kept frozen at -80°C until sectioning. A similar process was performed instilling and embedding in either 5% CMC or HPMC-PVP (Sigma; Cat. No. H8384 and PVP360, HPMC average molecular weight ∼22kDa, PVP average molecular weight Mn360). The latter was diluted 1:1 with 1x phosphate buffered saline (PBS) made in diethylpyrocarbonate (DEPC) treated water in order to reduce viscosity and allow even inflation of the lung lobe.

### Tissue preparation for MS-based omics

The blocked, embedded samples were shipped on dry ice from the University of Rochester Medical Center to Pacific Northwest National Laboratory and stored at -80°C until sectioned. The blocks were trimmed and mounted on a cryomicrotome chuck by freezing a small droplet of water, and then sectioned (12 μm; CryoStar NX70, Thermo Fisher) using a blade temperature of -16°C and specimen temperature of -18°C. Sections for MALDI-MSI were thaw-mounted onto indium tin oxide (ITO)-coated glass slides (Bruker Daltonics; Cat. No. 8237001) and placed in a desiccator (∼5 min) to bring to room temperature prior to matrix application. Serial sections were also placed on polyethylene naphthalate (PEN) membrane slides (Zeiss; Cat. No. 432301) and placed in a desiccator (∼5 min) to bring to room temperature for subsequent laser capture microdissection (LCM) analysis. Lastly, tissue was also collected (500 µm) in a 1.7 ml Sorenson tube (VWR; Cat. No. 53550–960) for MPLEx sample preparation as described below.

### MALDI-MSI sample preparation, data acquisition, and data processing

A M5 TM-Sprayer (HTX Technologies) was used for all MALDI matrix applications, as previously described (Veličković et al., 2020). For positive ion mode analysis, the matrix used was 2,5-dihydroxybenzoic acid (DHB; 40 mg/ml in 70% MeOH:H_2_O) (Sigma; Cat. No. 149357), with spraying parameters of 65–80°C nozzle temperature, a flow rate of 0.05 ml/min, 8–20 passes, a N_2_ pressure of 10 psi, a track spacing of 3 mm, and a 40 mm distance between the nozzle and sample was maintained for preparation of all samples. For negative ion mode analysis, the matrix used was N-(1-naphthyl-) ethylenediamine dihydrochloride (NEDC, 7 mg/ml in 70% MeOH:H_2_O) (Sigma; Cat. No. N9125), with spraying parameters of 65–80 °C nozzle temperature, a flow rate of 1.20 ml/min, 8–20 passes, a N_2_ pressure of 10 psi, a track spacing of 3 mm, and a 40 mm distance between the nozzle and sample was maintained for preparation of all samples. At least two replicates for each condition were analyzed.

MSI was performed on a 15 T MALDI-FTICR-MS (Bruker Daltonics) equipped with a SmartBeam II laser source (355 nm, 2 kHz) in positive and negative ion mode using 200 shots/pixel and a 35 µm pitch between pixels. FTICR-MS was externally calibrated using ESI Low Concentration Tuning Mix (Agilent; 5190–6895), and operated to collect 300–1,300 m*/z* using a 577 ms transient that translated to a mass resolving power of ∼170,000 at 400 m*/z*. The ion transfer and analyzer parameters remained constant based on optimizing the system using the ESI source to allow maximal transmission and detections of signals 600–900 m*/z*.

MALDI-FTICR-MS imaging data was imported into the SCiLS software (SQLite format, Bruker Daltonics) and converted into the imzML format with spectra restriction using only the *m/*z intervals of the imported peaks. The resulting imzML and ibd files were then uploaded to METASPACE (https://metaspace2020.eu) for molecular annotation and data visualization. The data was annotated using the SwissLipids database (Aimo et al., 2015) with a false discovery rate of 20% (Palmer et al., 2017). Annotated species with observed localization predominantly off-sample were filtered from the list. From METASPACE, CSV files were downloaded for each replicate, and annotations were cross-referenced to create a list consisting of the overlapping annotations. All the data generated in this study can be found at: https://metaspace2020.eu/project/lungmap-inflation-optimization. Pearson correlation coefficients of on- and off-tissue signals were determined in SCiLS. Briefly, for negative ion mode, PI (38:4) ([M-H]^-^, m/z = 885.5498) was used as the on-tissue signal, while m/z = 375.0698 was used as the off-tissue signal. For positive ion mode, PC (36:4) ([M + Na]^+^, m/z = 804.5514) was used as the on-tissue signal, while m/z = 550.3514 was used as the off-tissue signal.

### Bulk metabolomics, lipidomics and proteomics sample preparation

The MPLEx extraction procedure was adapted from the method of Folch et al. (Folch et al., 1957) by keeping the same final solvent proportions. However, the monophasic extraction step was not performed, as water was initially added to the sample along with the chloroform and methanol to simultaneously extract and partition molecules into the three different phases. Tissue lysates were resuspended in water, and four volumes of cold (-20°C) chloroform-methanol (2:1 [vol/vol]) solution was added to the samples. Samples were incubated for 5 min on ice, subjected to vortex mixing for 1 min, and centrifuged at 12,000 rpm for 10 min at 4°C. The upper aqueous phase and bottom organic phase, containing hydrophilic metabolites and hydrophobic lipids, respectively, were collected in glass autosampler vials. The interphases, containing proteins, were washed by adding 1 ml of cold (-20°C) methanol, vortex mixed for 1 min, and centrifuged at 12,000 rpm for 10 min at 4°C. The supernatants were discarded, and the resulting pellets were dried in a vacuum centrifuge for 5 min.

### Bulk metabolomics data acquisition and analysis

Chemical derivatization of extracted metabolites and subsequent gas chromatography-tandem mass spectrometry (GC-MS/MS) analysis were performed as reported previously (Kim et al., 2013). Briefly, dried metabolites were chemically modified with two derivatizations including methoxyamination and trimethylsilylation. The derivatized samples were analyzed by GC-MS, and the resulting data was processed with MetaboliteDetector (Hiller et al., 2009). Retention times were calculated based on a mixture of fatty acid methyl esters.

### Bulk lipidomics data acquisition and analysis

Samples were analyzed using liquid chromatography-tandem mass spectrometry (LC-MS/MS). Lipids were analyzed and identified as outlined by Kyle et al. (Kyle et al., 2017) Briefly, samples were dried *in vacuo* and reconstituted in 50 µl methanol, 10 µl of which was injected onto a reversed-phase Waters CSH column (3.0 mm × 150 mm × 1.7 µm particle size) connected to a Waters Acquity UPLC H class system interfaced with a Velos-ETD Orbitrap mass spectrometer. Lipid molecular species were separated over a 34 min gradient (mobile phase ACN/H_2_O (40:60) containing 10 mM ammonium acetate; mobile phase IACN/IPA (10:90) containing 10 mM ammonium acetate) at a flow rate of 250 μl/min. Samples were analyzed in both positive and negative ionization using HCD (higher-energy collision dissociation) and CID (collision-induced dissociation) to obtain high coverage of the lipidome. Confident lipid identifications were made using in-house developed identification software LIQUID (Kyle et al., 2017) where the tandem mass spectra were examined for diagnostic ion fragments along with associated hydrocarbon chain fragment information. To facilitate quantification of lipids, a reference database for lipids identified from the MS/MS data was created and features from each analysis were then aligned to the reference database based on their identification, m/z and retention time using MZmine 2 (Pluskal et al., 2010). Aligned features were manually verified and peak apex intensity values were exported for subsequent statistical analysis.

### Bulk proteomics data acquisition and analysis

MS analysis was performed using a QExactive mass spectrometer (Thermo Scientific, San Jose, CA) outfitted with a custom nano-electrospray ionization interface. Electrospray emitters were homemade using 150 μm o. d. × 20 μm i. d. chemically etched fused silica. The heated capillary temperature and spray voltage were 250 °C and 3.0 kV, respectively. Data was collected for 100 min following a 15 min delay from sample injection. Survey MS spectra were acquired from 400 to 2000 m/*z* at a mass resolution of 70 k (100 ms maximum accumulation time with 5 × 10^6^ automatic gain control (AGC) setting), while the top higher-energy collisional dissociation (HCD)-MS/MS spectra were acquired in data-dependent mode with an isolation window of 2.0 m/*z* and at a resolution of 17.5 k (100 ms maximum accumulation time with 1 × 10^5^ AGC setting) using a normalized collision energy of 32% and a 60 s exclusion time. For protein identification and quantification, the mass spectra data sets were analyzed using MaxQuant (v1.5.3.8) software. The mass spectra were searched against the UniProt protein sequence database (release 2015_04). Proteins were quantified using the label-free quantification (LFQ) approach. The peptide to spectrum matching and protein identification false discovery rate thresholds were set at 1%. Match between runs algorithm was applied during data analysis. The protein LFQ intensity data were extracted from the proteinGroups.txt file for protein quantification and analysis were performed using stats R package. Briefly, the proteins achieving 50% completeness among biological replicates in each condition were retained for quantification. Then the LFQ intensities for each condition were log2 transformed (log2-LFQ intensity values) and the missing values were imputed using the method reported by Tyanova et al. (Tyanova et al., 2016).

### Spatially resolved proteomics data acquisition and analysis

The tissue section on PEN membrane slides was washed and dehydrated by immersing the slide in a gradient of ethanol solutions for 30 s each change (70% EtOH, 95% EtOH, and 100% EtOH, respectively). The lung airway regions of interest, previously identified by MALDI-MSI, were dissected and collected in the corresponding well of the microPOTS chip preloaded with 3 µl of DMSO, which served as a capturing medium for the excised tissue sections. LCM was performed using a PALM MicroBeam system (Carl Zeiss MicroImaging, Munich, Germany) (Zhu et al., 2018; Xu et al., 2019). The microPOTS chip was covered and incubated at 75°C for an hour to dry DMSO solvent. Next, 2 µl of extraction buffer containing 0.2% dodecyl-β-D-maltoside (DDM), 0.5×PBS, 50 mM triethylammonium bicarbonate (TEAB), and 1 mM dithiothreitol (DTT) was dispensed to each well of the chip. The chip was incubated at 75°C for an hour. 0.5 µl of iodoacetamide (IAA) solution (10 mM IAA in 100 mM TEAB) was added to the corresponding wells with the samples, following the incubation step at room temperature for 30 min. All samples were subsequently digested by adding 0.5 µl of an enzyme mixture (10 ng of Lys-C and 40 ng of trypsin in 100 mM TEAB) and incubated the chip at 37°C for 10 h. Following digestion, peptides were acidified by adding 5% FA to each sample to the final 1% FA. Each sample was collected and dispensed into 30 µl aliquot of LC buffer A (water with 0.1% FA) then centrifuged at 10,000 rpm for 5 min, 25°C, and transferred ∼25 µl to an autosampler vial coated with 0.01% DDM. To minimize droplet evaporation, during every manipulation of the sample microPOTS chip was placed on an ice pack. Also, during each incubation, microPOTS chip was sealed with the chip cover and wrapped in aluminum foil and incubated in a humidified chamber.

Peptide mass analyses were performed using a QExactive Plus Orbitrap MS (Thermo Scientific) coupled with a custom LC system that consists of a PAL autosampler (CTC Analytics AG, Zwingen, Switzerland), two Cheminert six-port injection valves (Valco Instruments, Houston, United States), a binary nanoUPLC pump (Dionex UltiMate 3000; Thermo Scientific), and a HPLC sample loading pump (1200 Series; Agilent, Santa Clara, United States). Sample was fully injected into a 25 µL loop and loaded onto an SPE precolumn (150 µm i. d, 5 cm length) using Buffer A at a flow rate of 3 μL/min for 30 min. Following SPE clean-up, the concentrated sample was backflushed on an LC column (50 µm i. d, 60-cm Self-Pack PicoFrit column, New Objective, Woburn, United States). Both SPE precolumn and column were slurry-packed with 5-µm and 3-µm Jupiter C18 packing material (300-Å pore size) (Phenomenex, Terrence, United States), respectively. Chromatographic separation was performed at 200 nL/min using the following gradient: 1–8% (2.6–12.6 min), 8–25% (12.6–107 min), 25–75% (107–122.6 min), and 75–95% (122.6–125.9 min) of Buffer B (0.1% formic acid in acetonitrile) followed by column washing and re-equilibration. Separated peptides were introduced to the ionization source in which high voltage (2200 V) was applied to generate electrospray and ionize peptides. The ion transfer tube was heated to 300 °C and the S-Lens RF level was set to 60. Full MS scan was acquired across scan range of 300 to 1,800 m/z at a resolution of 70,000, combined with a maximum injection time of 20 ms and AGC target value of 3e6. Twelve data-dependent MS/MS scans were recorded per MS scan, at a resolving power of 17,500 combined with a maximum injection time of 50 ms and AGC target value of 1e5, with an isolation window of 2 m/z.

The raw data collected during the LC-MS/MS analyses were processed using MaxQuant (v1.6.0.16) software. The mass spectra were searched against the UniProt protein sequence database (release 2022_10). Proteins were quantified using the label-free quantification (iBAQ) approach. The peptide to spectrum matching and protein identification false discovery rate thresholds were set at 1%. Two peptides minimum were required for protein identification. Match between runs algorithm was applied during data analysis. The instrument raw files were deposited on MassIVE (MassIVE accession: MSV000090561) and the code to analyze the data was uploaded on GitHub (https://github.com/GeremyClair/MALDI_informed_microdissected_Airway_proteomics). The complete description of MaxQuant settings is provided as the mqpar. xml file. The protein iBAQ intensity data were extracted from the proteinGroups.txt file. The proteins achieving 60% completeness among the microdissected areas were retained. Then the iBAQ intensities for each condition were log2 transformed (log2-LFQ intensity values) and median centered. Enrichment analysis were performed using enrichR (Xie et al., 2021).

## Results

Lung inflation is needed prior to structural and molecular imaging (e.g., histological, MSI) experiments to maintain tissue architecture, as this is the only way to reveal the variety of fine structures and the location of endogenous molecules in an *in vivo-*like context. A wide variety of sample preparation parameters can influence the quality of data obtained from multi- and spatial omics experiments. Here, a particular emphasis was put on investigating agarose inflation, as it had been reported to be compatible with micro-CT scans after inflation, something that is desired for future multi-modal imaging (including molecular tomography) experiments and to maximize use of rare human lung samples. We optimized conditions for tissue preparation and handling of bulk and spatial proteomics, lipidomics, and metabolomics analyses for agarose inflated human lung.

### Bulk and spatially resolved omics of agarose inflated lung blocks

Bulk omics experiments contribute a critical output in terms of the molecular information about the tissue, and they also aided in confirming molecular annotations that are detected in the MALDI-MSI analysis using METASPACE. METASPACE, a publicly available web-based platform, enables metabolite annotation in an FDR confidence-controlled manner for MSI experiments. To generate unbiased bulk proteomics, lipidomics, and metabolomics profiling on the same tissue block as the ones employed for MALDI-MS imaging, ∼500 um of the agarose inflated lung blocks were sectioned and the biomolecules were extracted following the MPLEx procedure. The proteins, which were digested into peptides, and the lipid fractions of the extraction were analyzed by LC-MS/MS, while the metabolomic fraction was analyzed by GC-MS/MS. The number of species found in the bulk omics experiments indicates that agarose inflation was amendable for detecting many different biomolecules using our different bulk omics assays (2518 ± 184 proteins, 405 ± 34 lipids, and 173 ± 18 metabolites; Table 1). Of note, we detected several airway-specific proteins to be present within our sample from the bulk proteomics analyses.

**TABLE 1:** Number of species identified from three samples from LC-MS/MS proteomics and lipidomics, and GC-MS/MS metabolomics from agarose inflated 5% CMC embedded human lung. Tables containing the identifications of the species can be found in the supplemental information. Lipid species identified are from positive and negative ion mode combined.

Omics technique	Number of confidently identified species
LC-MS/MS Proteomics	2518 ± 184
LC-MS/MS Lipidomics	405 ± 34
GC-MS/MS Metabolomics	173 ± 18

To confirm that these potential airway-specific proteins were localized to these specific regions, we isolated cells from airway structures in a serial tissue section using laser capture microdissection (LCM). Eight airway regions were successfully collected by LCM and then analyzed by a microPOTS (Processing in One pot for Trace Samples) proteomic analysis (Zhu et al., 2018; Xu et al., 2019). The areas collected can be seen in Supplementary Figure S1 and were determined by our MALDI-MSI analysis. In at least five of the microdissected regions, 2118 proteins were consistently detected, several of which were airway-specific proteins (e.g., SCGB1A1, RSPH1, TACSTD2, GRP, and MYH11) and about 5.8% of these proteins (123 were matrix proteins. We used enrichR (Xie et al., 2021) to identify the cell-types specific proteins in the sections. Enrichments against the HubMAP ASCT plus B tables (https://hubmapconsortium.github.io/ccf-asct-reporter/) revealed that proteins specific to airway smooth muscle cells were the most enriched (q-value <1E-17). The sections also seemed to contain, lung matrix fibroblast 2, pulmonary endothelial cells, and bronchial ciliated cells. This LCM experiment allows for a closer examination of the proteomic composition of cells located in the airway regions isolated and starts to allow for some co-localization comparisons to be made between the proteome and the metabolome.

**FIGURE 1:**
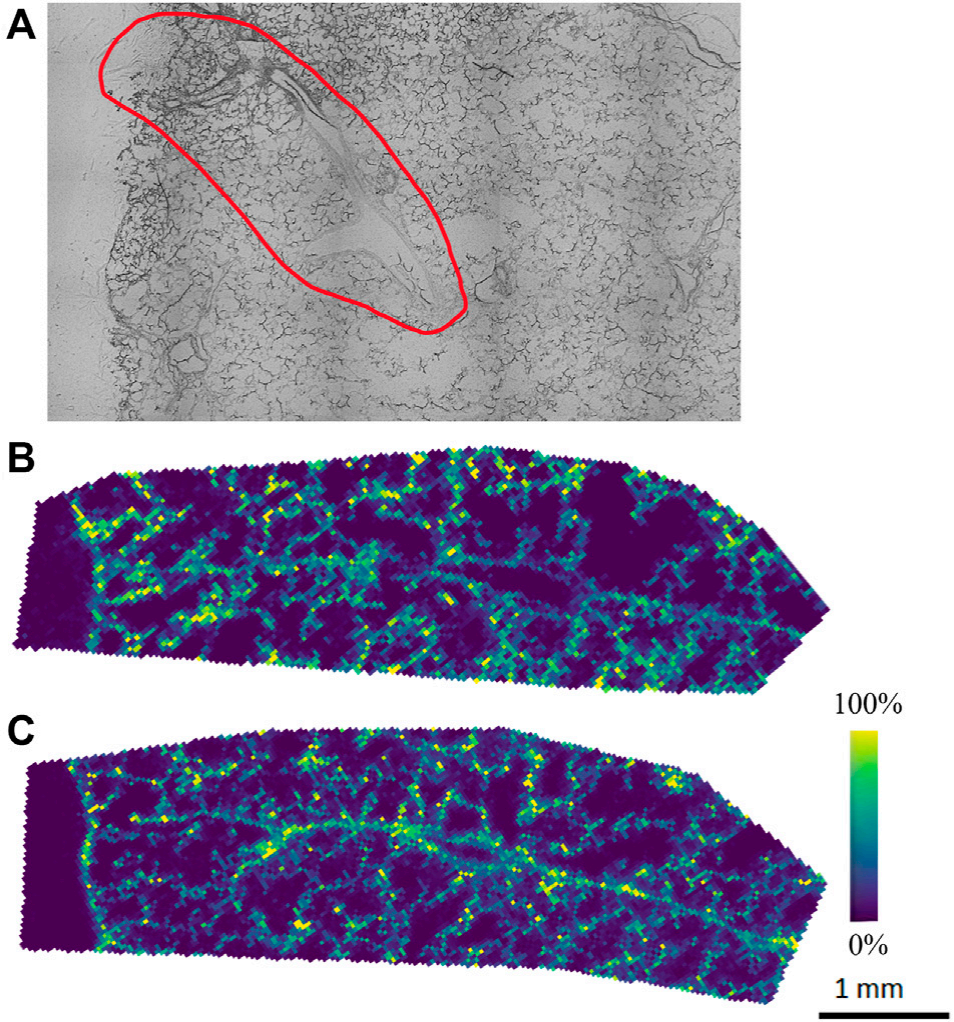
Representative **(A)** optical image and ion images from **(B)** initial positive and **(C)** negative ion mode analysis of agarose inflated 5% CMC embedded lung, where purple indicates no presence of the species and yellow indicates high abundance of the species.

The agarose inflated lung block was sectioned, and tissue sections were thaw-mounted onto ITO slides to be analyzed by MALDI-MSI to explore the lipid and metabolite profile. Alternate serial sections were placed on two different slides to be analyzed in both positive and negative ion mode to obtain the most comprehensive molecular coverage. In this analysis, we chose a specific region of interest within the lung tissue, based on brightfield optical microscopy mapping of the tissue sections, which contained both distal parenchyma and airways. For positive and negative ion mode analysis, DHB and NEDC matrices were used to aid in ionization and detection of molecules, respectively. Figure 1 illustrates representative ion images for both the positive ion and negative ion mode analysis. Data from the MALDI-MSI analysis was then uploaded to METASPACE and putative identifications were initially made using the SwissLipids database at a 20% FDR (Aimo et al., 2015; Palmer et al., 2017). In positive ion mode 269 ± 23 species were identified and in negative ion mode 158 ± 18 were identified. Bulk lipidomic data was used to provide more confident annotations to these putative identifications.

Our initial MALDI-MSI results with METASPACE show molecular species endogenous to the lung tissue with different spatial distributions. However, the number of putative annotations was relatively low, especially in the negative ion mode analysis (158 ± 18), which we attribute to the molecular makeup of the agarose itself, which could hinder ionization in negative ion mode. As such, a systematic optimization approach was performed to improve the MALDI matrix spraying parameters, as this is a critical step in the sample preparation process for MALDI-MSI analysis. As noted earlier, MALDI-MSI relies on a crystallized layer of energy-absorbing matrix molecules over a sample. In MALDI-MSI, homogeneous matrix deposition is important to ensure that any detected structure or heterogeneity in an ion image reflects the actual molecular content of the sample, rather than matrix application artifacts (e.g., “hot spots”) (Huizing et al., 2019). Additionally, analyte delocalization, which is caused by the diffusion of endogenous compounds inside the matrix solution before crystallization, needs to be minimized to retain the spatial-molecular features of the sample being analyzed (Veličković et al., 2020). By optimizing the matrix spraying parameters specifically for an agarose inflated sample, we aimed to increase the number of detected, and thus annotatable, species for both positive and negative ion mode analyses.

### Optimization of matrix coating parameters for agarose inflated lung tissue

**FIGURE 2:**
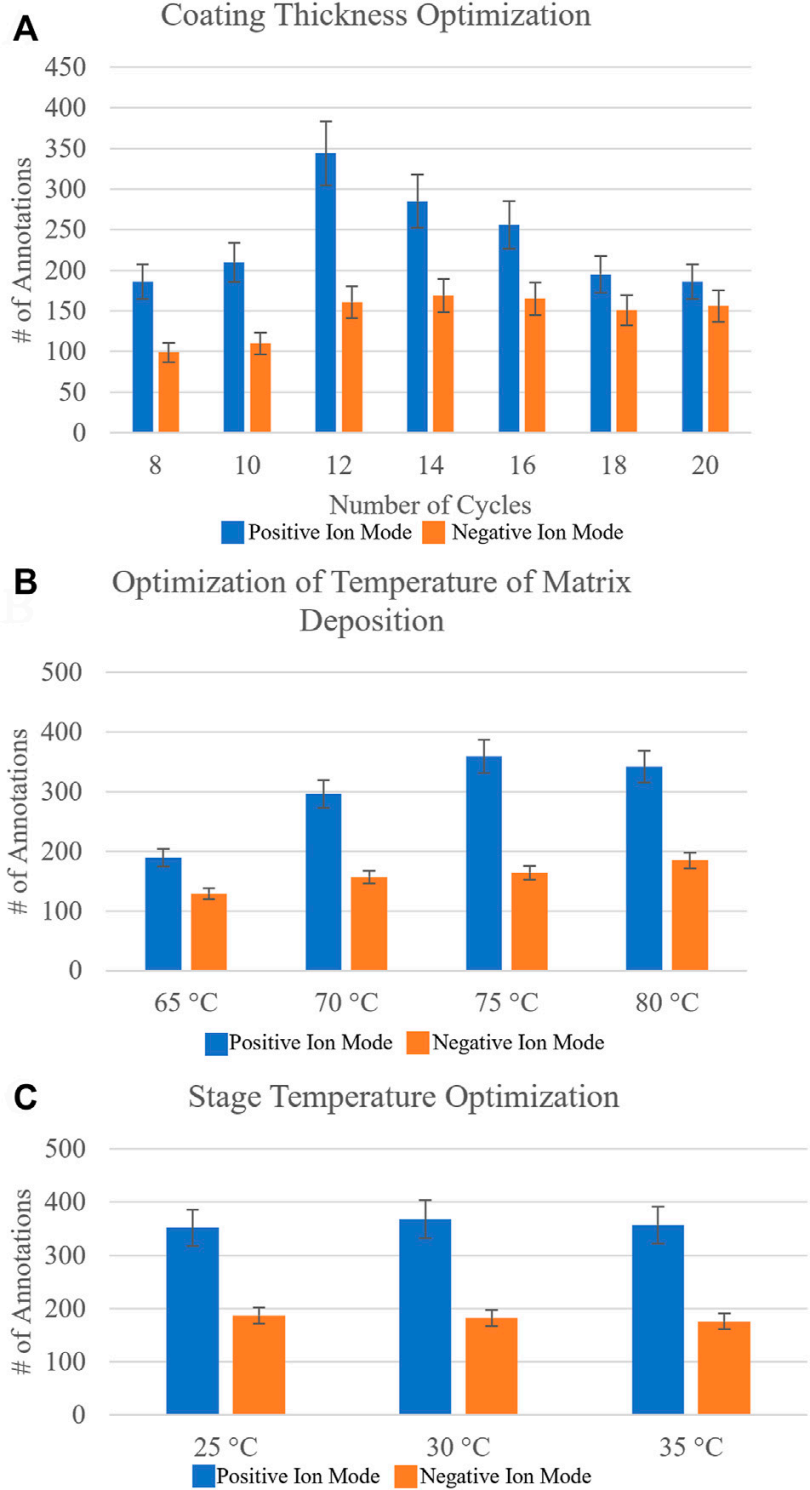
Systematic optimization of matrix deposition for positive and negative ion mode MALDI-MSI of agarose inflated 5% CMC embedded human lung samples. Homogeneous matrix deposition is important to ensure the detected ion image distribution reflects the actual molecular content of the sample, rather than matrix application artifacts. Analyte delocalization should be minimized to retain the spatially-specific molecular features of the sample being analyzed. Optimized matrix deposition was determined by looking at the number of resulting annotations in METASPACE using the SwissLipids database at 20% FDR and Pearson’s correlation coefficients of an on-tissue signal and an off-tissue signal (Supplementary Table S1). **(A)** The number of resulting annotations for increasing the number of passes of DHB matrix (which is proportional to concentration) for positive ion mode and NEDC matrix for negative ion mode (blue and orange, respectively). Based on reproducible number of annotations it was found for DHB 12 passes was optimal and for NEDC 14 passes was optimal. **(B)** The resulting number of annotations by varying the temperature of matrix deposition at the nozzle from 65°C to 80°C. It was found that for positive ion mode with DHB, 75°C was optimal, whereas for negative ion mode with NEDC, 80°C was optimal. **(C)** The resulting number of annotations from increasing the stage temperature that the sample slide sits on from 25°C to 35°C. For DHB the ideal stage temperature was found to be 30°C, while for NEDC the ideal stage temperature was determined to be 25°C. Errors bars represent coefficient of variance percentage for each condition.

A sequential approach rather than a randomized approach was taken to optimize the matrix spraying parameters. The number of passes of matrix applied to the sample was optimized first. This parameter directly affects the matrix density on the sample, as well as the crystal sizes that can boost or limit ionization efficiencies. A range of 8–20 passes, testing even pass numbers, was investigated on three different sections from three different biological replicates, and the data were uploaded to METASPACE. Initial putative identifications were made using the SwissLipids database at 20% FDR. To determine the identification of the lipid isomer from our MALDI-MSI data, we utilized the bulk lipidomics data generated from serial sections, which provided higher confidence molecular annotations, as the head group and fatty acid chains could now be verified from ion fragmentation with MS2, for example. Figure 2A shows the number of annotations found for each investigated pass of matrix applied. It was found that for negative ion mode with NEDC matrix, 14 passes (11.47% CV) was optimal, while for positive ion mode with DHB matrix the optimal number of passes was 12 (8.55% CV), based on where the number of reproducible and confirmed annotations was the highest.

The total number of annotations is only one parameter for testing optimal matrix application conditions. Often an increase in sensitivity can lead to an increase in molecular delocalization of species, where using too ‘wet’ of spraying conditions can increase extraction of endogenous molecules but cause ‘smearing’ of location specific molecules (Veličković et al., 2020). We calculated the Pearson’s correlation coefficients of specific ion images to provide a direct metric of molecular delocalization of endogenous species (Veličković et al., 2020). Specifically, the two ion signals used were not colocalized, or rather were anti-correlated with each other. We used an on-tissue ion signal and the other was an off-tissue ion signal (Veličković et al., 2020). For the correlation coefficients for negative ion mode, PI (38:4) ([M-H]^-^, m/z = 885.5498) was used as the on-tissue signal, while m/z = 375.0698 was used as the off-tissue signal. For positive ion mode, PC (36:4) ([M + Na]^+^, m/z = 804.5514) was used as the on-tissue signal while m/z = 550.3514 was used as the off-tissue signal. Off-tissue signals can be traced back to the molecular makeup of the agarose inflation media which contains varies sugar compounds. Here, the smaller the Pearson’s correlation coefficient between the two ion images, the smaller the observed molecular delocalization. These Pearson’s correlation coefficients (Supplementary Table S1) confirmed what we observed from the MALDI-MSI images, where 14 passes of NEDC matrix and 12 passes of DHB matrix provided the best results for negative and positive ion mode analysis, respectively.

The next matrix spraying parameter tested for optimization was the temperature of the matrix deposition at the nozzle of the sprayer. Increasing the temperature at which the matrix solvent is applied to the sample will reduce the droplet size of the matrix mist, and will thus generate smaller matrix crystals, but at the cost of creating a less efficient extraction. For this we tested a range of nozzle temperatures from 65°C to 80°C, testing in 5°C increments. Figure 2B shows the resulting number of annotations from METASPACE using the SwissLipids database (20% FDR) for these optimization experiments. For negative ion mode, it was found the optimal temperature of matrix deposition at the nozzle to be 80°C (10.71% CV), and for positive ion mode, 75°C (12.38% CV) was determined to be the optimal temperature. Pearson’s correlation coefficients (Supplementary Table S1) again provided another metric for determining optimized conditions, as the two ion signals on- and off-tissue investigated showed the least amount of molecular delocalization under these conditions.

Lastly, we investigated changing the stage temperature where the ITO-coated glass slide-mounted tissues are placed for matrix application. We tested stage temperatures from 25°C to 35°C, in 5°C increments. Figure 2C highlights the number of annotations found for this final sequential optimization step. For negative ion mode, we found the stage temperature had no measurable effect on the number of annotations obtained. For positive ion mode, a 30°C (10.69% CV) stage temperature provided the highest number of annotations, but the difference was not statistically significant. Representative ion images for the conditions tested can be found in Supplementary Figure S2.

**FIGURE 3:**
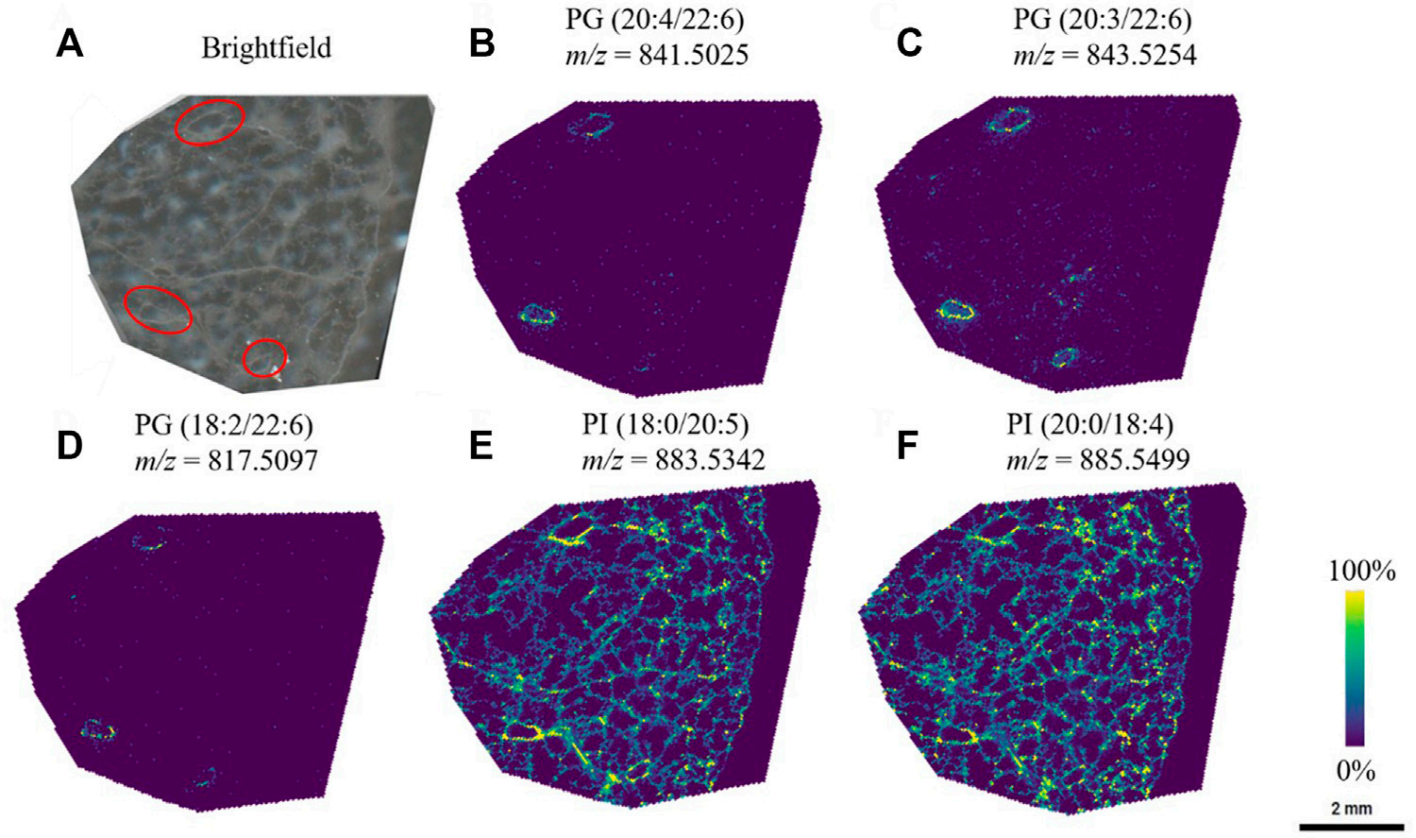
PG lipid species were observed to be localized to a specific region (red circles) in the lung, whereas other species were visualized throughout the lung tissue. **(A)** Brightfield image of the lung tissue section, and the corresponding ion images of **(B)** PG (20:4/22:6) **(C)** PG (20:3/22:6) **(D)** PG (18:2/22:6) **(E)** PI (18:0/20:5), and **(F)** PI (20:0/18:4). Lipid species were first identified through METASPACE and confirmed by LC-MS/MS *via* analysis of the bulk tissue extract.

Through systematic optimization of the matrix spraying parameters for MALDI-MSI analysis for agarose inflated lung blocks, we could confidently observe unique lipid localizations within the airway structures in the lung tissue sections. Particularly in the negative ion mode analysis, we detected several phosphatidylglycerol lipids (PGs) that showed a high abundance and specificity to the airways in the sample (Figure 3). An interesting characteristic of the PGs were that most of them contained 22:6 fatty acid chains (confirmed through the bulk lipidomics experiments). PGs are typically present at a level of 1–2% in most mammalian tissue, but PGs are known to be the second most abundant phospholipid subclass in mature lung surfactant (Dexter et al., 2019). Here, we identified that those containing 22:6 fatty acids preferentially localized in airways. Bulk lipidomic analysis confirmed the presence of several PG 22:6 lipid species within the tissue samples, which permitted us to confirm our putative identification obtained from METASPACE for our MALDI-MSI experiments. These and similar molecular lipid species of PG containing 22:6 fatty acids have also been previously reported in bulk and cell sorted lung tissue (Dautel et al., 2017; Kyle et al., 2018).

### Initial exploration of alternative inflation and embedding media

In addition to testing agarose inflation and 5% CMC embedding, comparisons were made to 5% CMC and HPMC-PVP inflation and embedding, as these are other popular inflation methods used within the field. These blocks were prepared using the same methods and they were analyzed by MALDI-MSI and bulk omics-based analyses, as detailed above. We found the 5% CMC inflated and embedded samples very difficult to cryosection in comparison to the other two embedding medias, even with varying the blade and specimen holder temperature. We observed that the CMC inflation made lung tissue blocks, inherently composed of more CMC than tissue, prone to shattering by the blade and very difficult to obtain full sections of the lung tissue free of artifactual tears or other distortions (Supplementary Figure S3). On the other hand, the HPMC-PVP sample was very similar to the agarose inflated sample in terms of sectioning and whole tissue sections could easily be obtained without tears or other distortions. From MALDI-MSI and bulk omics analyses, we found a similar number (within the margin of error of our agarose data) of annotations was obtained for each of these samples in comparison to the agarose inflated sample (Supplementary Table S2). A comparison of lipids per subclass identified in the three inflation media from bulk lipidomics can be found in Supplementary Table S3.

The 5% CMC inflated and embedded sample and the HPMC-PVP sample were then prepped for bulk omics experiments. We found a limitation to using the MPLEx method for HPMC-PVP tissue, as the lipid fraction crashed out of solution and created a gel-like paste that could not be redissolved for analysis by LC-MS/MS. Given the limited sample availability to re-test MPLEx, we chose to test the methyl-tert-butyl ether (MTBE) extraction method (Matyash et al., 2008) to isolate lipids from the HPMC-PVP sample. The resulting species found from the proteomics, lipidomics, and metabolomics experiments can be seen in Supplementary Table S2. All three inflation and embedding methods investigated produced a similar number of species detected and annotated. Especially in the context that we could only run one sample of each tissue due to the nature of collecting these precious tissue samples. This indicated to us that each of these inflation methods do indeed have their own merit, but ultimately had notable limitations when performing multi-modal bulk and spatial omics experiments.

## Discussion

Within the bulk proteomics data, we found several airway specific proteins to be present within our sample. This highlights the advantages of using multiple analysis techniques to gain a deeper insight into a single sample on multiple different phenotypic levels. One disadvantage to the bulk lipidomic and proteomic results discussed here is they do not give any spatial information of where species are located within the tissue. To identify potential proteins that may specifically correlate with the PG spatial localization we could see with MALDI-MSI, LCM was utilized to specifically isolate and analyze these airway structures. Importantly, numerous markers of airway epithelial cells (Sun et al., 2022) were detected in the microdissected regions including: the well-described marker of secretory cells uteroglobin (SCGB1A1) (Zhu et al., 2019); RSPH1 that localizes within the cilia of airway epithelial cells (Kott et al., 2013); the recently described basal marker TACSTD2 (Lenárt et al., 2021; Sun et al., 2022), the neuroendocrine marker GRP (Wang and Conlon, 1993), the myoepithelial cell marker MYH11 (Anderson et al., 2017) and the serous cell markers LYZ (Yu et al., 2022)and LTF (Travaglini et al., 2020). The data showed the presence of surfactant proteins (SP). While SP-A and SP-B were detected in most of the microdissected tissues, SP-C was not. Notably, SP-C is the only SP known to be solely produced by Alveolar Type 2 (AT2) cells (Jacob et al., 2017). Further enrichment analysis performed in DAVID for the protein detected in these regions revealed an enrichment of cilia related Gene Ontology terms such as ‘ciliary tip’ (GO:0097542), ‘ciliary base’ (GO:0097546), and ‘intraciliary transport’ (GO:0042073). Overall, these results suggest that, as intended, the microdissection enriched for airway tissue that can be analyzed by proteomics.

In MALDI-MSI, homogeneous matrix deposition is important to ensure that any detected structure or heterogeneity in an ion image reflects the actual molecular content of the sample, rather than matrix application artifacts (e.g., “hot spots”). Additionally, analyte delocalization, which is caused by the diffusion of endogenous compounds inside the matrix solution before crystallization, should be minimized to retain the spatial-molecular features of the sample being analyzed. Optimized matrix deposition was determined by looking at the number of resulting annotations and Pearson’s correlation coefficients of an on-tissue signal and an off-tissue signal. For low signal delocalization a small Pearson’s correlation coefficient is desired. In Figure 2, we illustrate how different spraying parameters effected our MALDI-MSI sensitivity (by using number of annotations as a metric), and we determine how these spraying parameters effected molecular delocalization (the Pearson’s correlation coefficients for all conditions tested can be found in Supplementary Table S1).

There is an advantage to identifying a singular method for inflation preparation of human lung to maintain microstructural and cellular integrity, while making the tissue accessible to multiple methods of molecular analysis and experimental manipulation. Also, having a method that may be used in series to assess more thoroughly the micro-environments within the multiple lobes composing the dual lung organ is ideal. Overall, with our multi-technique experiment of LCM proteomics and MALDI-MSI-based metabolomics and lipidomics, we illustrated that ability to make spatial comparisons between the proteome and the metabolome in the human lung.

## Conclusion

Since diseases are biomolecularly multifaceted and it is important to understand them on multiple physiochemical levels. Mass spectrometry approaches can offer in-depth detection of a wide range of biomolecules from a single sample, and complementary information obtained from spatial multi-omics approaches can provide a comprehensive understanding of the biomolecular state within cells and anatomical features of tissues. The agarose inflated sample proved to be a suitable inflation method holistically for multi-omic analysis. Through a systematic optimization process of the matrix spraying parameters for MALDI-MSI analysis in both positive and negative ion mode, the number of confirmed molecular annotations were boosted, and we were able to start confidently identifying unique localizations of lipids to the airways present in the tissue section, which we could correlate with spatial proteomics data. In the future, we plan to use the methods described here to explore the biomolecular signatures present in both diseased and healthy lung samples.

## Compliance with ethical standards

Donor lung samples, authorized for research, were provided through the federal United Network of Organ Sharing *via* National Disease Research Interchange (NDRI) and International Institute for Advancement of Medicine (IIAM) and entered into the NHLBI LungMAP BioRepository for INvestigations of Diseases of the Lung (BRINDL) at the University of Rochester Medical Center as de-identified, non-human-subjects research overseen by the IRB as RSRB00047606, as previously described (Ardini-Poleske et al., 2017; Bandyopadhyay et al., 2018).

## Data Availability

The datasets presented in this study can be found in online repositories. The names of the repository/repositories and accession number(s) can be found in the article/Supplementary Material. Additional data supplementary files can be found here: https://doi.org/10.6084/m9.figshare.19295387.v1.
