# Supplementary material for: An optimized approach and inflation media for obtaining complimentary mass spectrometry-based omics data from human lung tissue
Source: Front Mol Biosci. 2022 Nov 16;9:1022775. doi: 10.3389/fmolb.2022.1022775 (PMC9709465; doi:10.3389/fmolb.2022.1022775)
Supplement: Supplementary file 1 [file Table1.DOCX]

**Supplemental Information For:**

**An optimized approach for obtaining complimentary mass spectrometry-based omics data from human lung tissue**

Jessica K. Lukowski^1^, Heather Olson^1^, Marija Velickovic^1^, Juan Wang^1^, Jennifer E. Kyle^1^, Young-Mo Kim^1^, Sarah M. Williams^1^, Ying Zhu^1^, Heidie Huyck^2^, Matthew D. McGraw^2^, Cory Poole^2^, Lisa Rogers^2^, Ravi Misra^2^, Theodore Alexandrov^3^, Charles Ansong^1^, Gloria Pryhuber^2^, Geremy Clair^1^, Joshua N. Adkins^1^, James Carson^4^, Christopher R. Anderton*^1^

^1^Pacific Northwest National Laboratory (PNNL), Richland, WA, USA

^2^University of Rochester Medical Center, Rochester, NY, USA

^3^Structural and Computational Biology Unit, European Molecular Biology Laboratory, Heidelberg, Germany

^4^Texas Advanced Computing Center (TACC), University of Texas at Austin, Austin, TX, USA

* Christopher.Anderton@pnnl.gov; 902 Battelle Boulevard, Richland, Washington 99352; 509-371-7970

**Table of Contents:**

Figure S1: LCM analysis regions and resulting proteomic identifications……………………………….….2

Figure S2: Resulting MALDI-MS images from matrix deposition optimization………………...……..….3

Figure S3: Images of tissue sections of agarose, 5% CMC, and HPMC inflated samples...............….……..4

Table S1: Pearson’s correlation coefficients for matrix deposition optimization…………………………..6

Table S2: Resulting annotations from 5% CMC and HPMC inflated samples………………………….…7

Table S3: Number of lipids per subclass identified in agarose, 5% CMC, and HPMC inflated samples.....8

Excel file 1: Bulk metabolomics results for all samples analyzed

Excel file 2: Bulk and LCM proteomics results for all samples analyzed

Excel file 3: Bulk lipidomics results for all samples analyzed


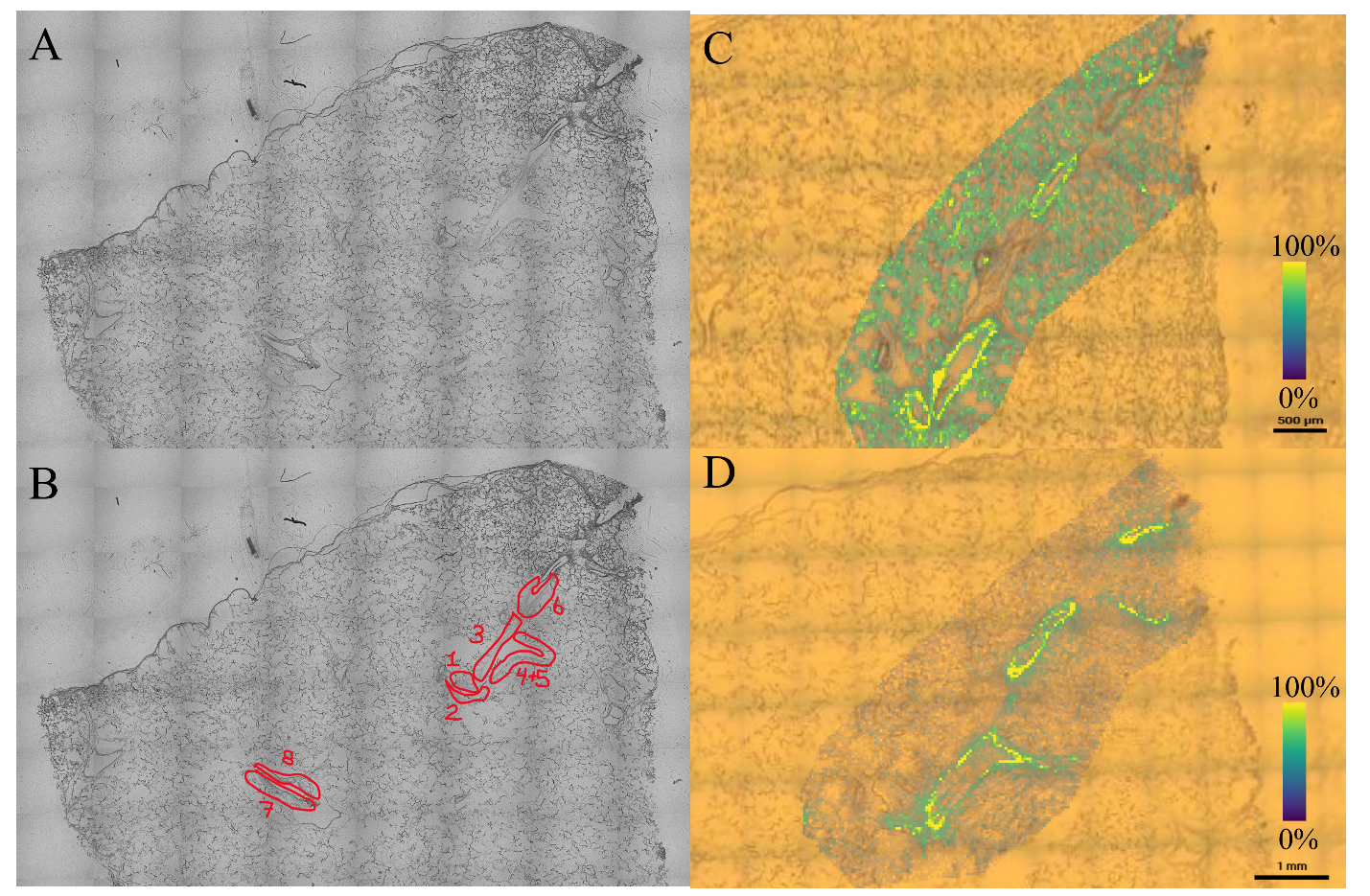


**Supplemental Figure 1.** Areas isolated for the MALDI-MSI guided laser capture microdissection (LCM)-based proteomics analysis. (A) Brightfield image of the lung tissue section before LCM. (B) Areas isolated around the airways. (C) PC (18:1/18:0) lipid distribution found in positive ion mode MALDI-MSI analysis of a serial section of tissue, and (D) PG (16:0/22:6) lipid distribution found in negative ion mode MALDI-MSI analysis of a serial section of tissue. Oure MALDI-MSI results show that these areas showed high intensity of several lipid species, but of particular interest was that the PG (22:6) lipid species eemed to be highly abundance solely in the airway structures. A microPOTS proteomic analysis was performed to identify the proteins specifically present in the airway structures and the results were pooled together. Protein identifications can be found in the supporting excel document.

**
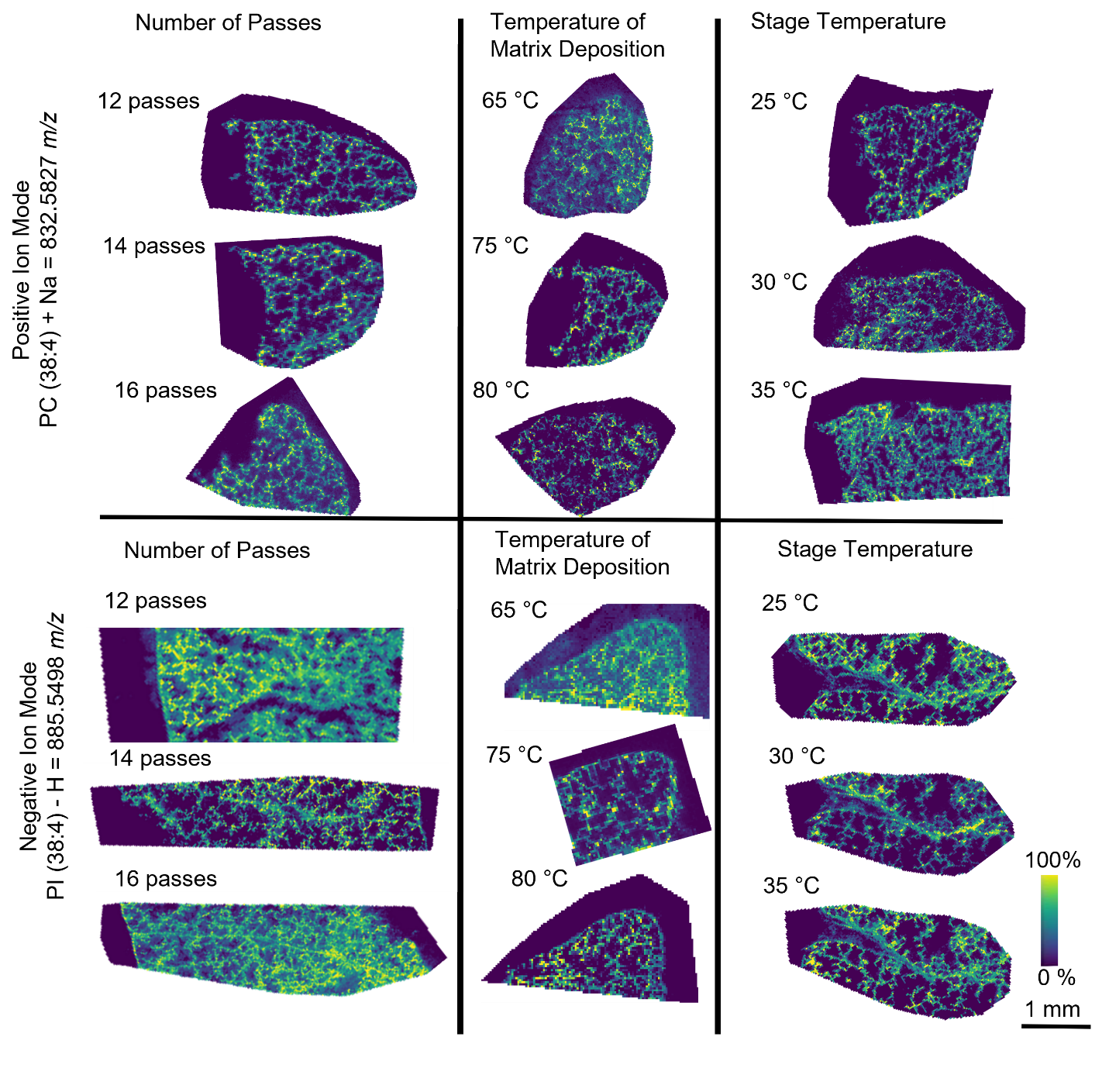
Supplemental Figure 2.** Example MALDI-MS ion image data from the optimization of matrix deposition for both positive and negative ion mode. In MALDI-MSI, homogeneous matrix deposition is important to ensure that any detected structure or heterogeneity in an ion image reflects the actual molecular content of the sample, rather than an artifact from matrix application. Additionally, analyte delocalization, which is caused by the diffusion of endogenous compounds inside the matrix solution before crystallization, should be minimized to retain the spatial-molecular features of the sample being analyzed. Optimized matrix deposition was determined by looking at the number of resulting annotations and Pearson’s correlation coefficients between an on-tissue signal and an off-tissue signal. For low signal delocalization, a small Pearson’s correlation coefficient is desired. For example, (bottom row, middle column) in negative ion mode analysis, a temperature of matrix deposition at 65 °C, the ion image appears fuzzy. This indicates signal delocalization, especially in comparison to ion image of the sample where the matrix that was deposited at 80 °C. This image sharpness was verified through measurement of Pearson’s correlation coefficients (Supplemental Table 1).


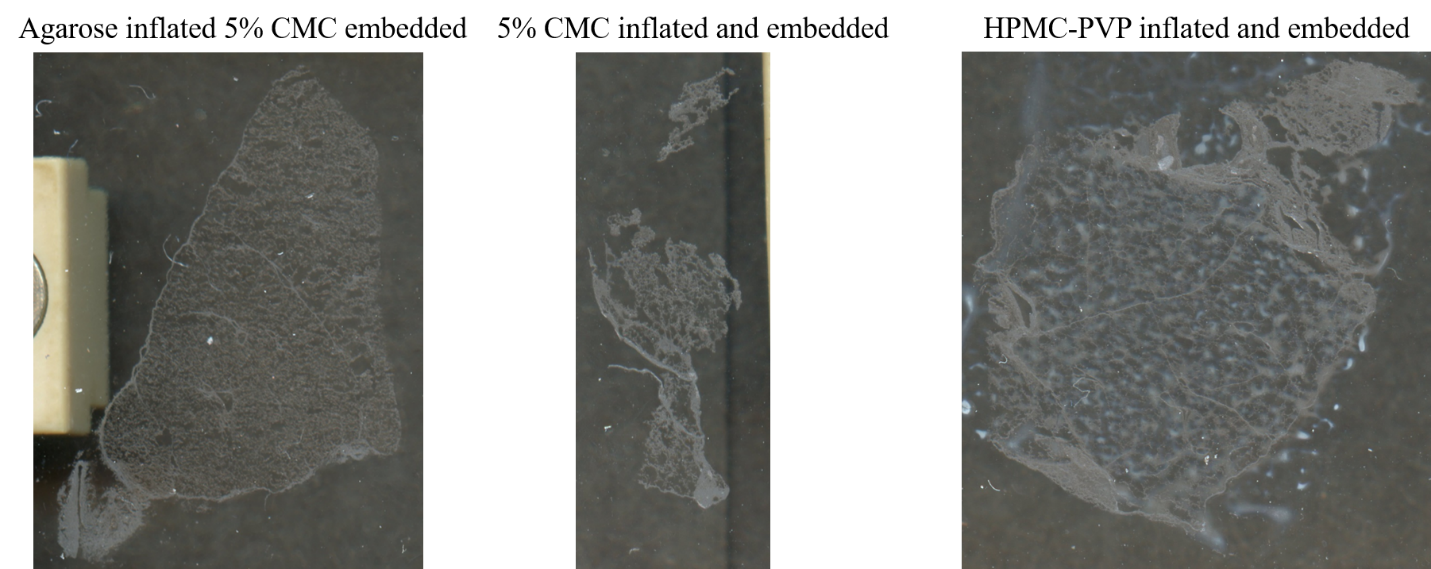


**Supplemental Figure 3**. Optical micrographs of sections obtained from agarose, 5% CMC, and HPMC-PVP inflated samples. Agarose and HPMC-PVP samples sectioned with ease, while the 5% CMC inflated sample was difficult to obtain a whole section from.

**Supplemental Table 1.** Pearson’s correlation coefficients for each condition tested during matrix optimization. For negative ion mode, m/z = 885.5498 (PI (38:4, [M-H]^-^) was used as the on-tissue signal, while m/z = 401.1542 was used as the off-tissue signal. For positive ion mode, m/z= 804.5514 (PC (36:4), [M+Na]^+^) was used as the on-tissue signal, while m/z= 550.3514 was used as the off-tissue signal.


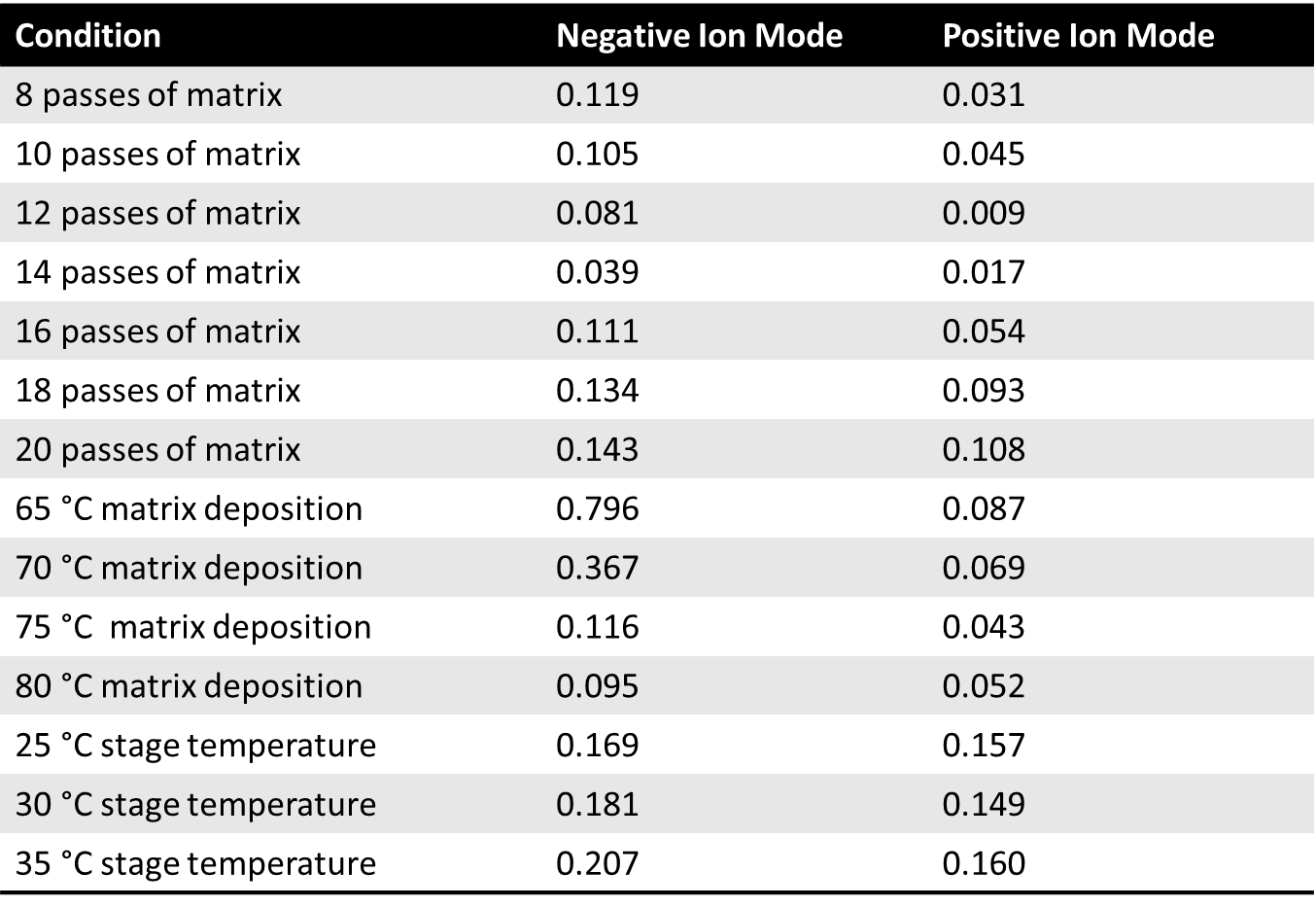


**Supplemental Table 2:** Resulting number of identifications found, as described in the Methods section, for 5% CMC (n=1) and HPMC-PVP (n=1) inflated samples via bulk omics and MALDI-MSI.

| Omics Technique | 5% CMC Inflated | HPMC-PVP |
| --- | --- | --- |
| LC-MS/MS Proteomics | 3441 | 2960 |
| LC-MS/MS Lipidomics  (Positive and Negative Ion Mode) | 486 | 346 |
| GC-MS/MS Metabolomics | 198 | 229 |
| MALDI-MSI Positive Ion Mode | 290 | 279 |
| MALDI-MSI Negative Ion Mode | 170 | 189 |

**Supplemental Table 3**: Number of unique lipid classes identified from bulk lipidomics for each of the inflated lung blocks tested.

|  | Number of lipids identified | | |
| --- | --- | --- | --- |
| Subclass | **Agarose** | **5% CMC** | **HPMC** |
| Carnitine (Carn) | 5 ± 1 | 5 | 3 |
| Cholesteryl ester (CE) | 9 ± 4 | 2 | 2 |
| Ceramide (Cer) | 17 ± 1 | 19 | 15 |
| Hexosylceramide (HexCer) | 0 | 4 | 2 |
| Dihexosylceramide (Hex2Cer) | 0 | 0 | 0 |
| Sphingomyelin (SM) | 32 ± 3 | 26 | 29 |
| Phosphatidic Acid (PA) | 0 | 0 | 1 |
| Cardiolipin (CL) | 0 | 7 | 2 |
| Lysophosphatidylcholine (LPC) | 19 ± 2 | 16 | 16 |
| Phosphatidylcholine (PC) | 72 ± 4 | 85 | 55 |
| Oxidixed phosphatidylcholine (PCO) | 8 ± 1 | 8 | 11 |
| Phosphorylated (phosphatidylcholine) PCP | 2 ± 1 | 5 | 2 |
| Lysophosphatidylethanolamine (LPE) | 17 ± 2 | 17 | 15 |
| Phosphatidylethanolamine (PE) | 32 ± 4 | 46 | 17 |
| Oxidized Phosphatidylethanolamine (PEO) | 6 ± 2 | 3 | 3 |
| Phosphorylated Phosphatidylethanolamine (PEP) | 1 ± 1 | 35 | 5 |
| Lysophosphatidylglycerol (LPG) | 9 ± 3 | 5 | 7 |
| Phosphatidylglycerol (PG) | 38 ± 7 | 56 | 37 |
| Lysophosphatidylinositol (LPI) | 7 ± 2 | 8 | 8 |
| Phosphatidylinositol (PI) | 26 ± 3 | 29 | 30 |
| Lysophophatidylserine (LPS) | 4 ± 2 | 1 | 1 |
| Phosphatidylserine (PS) | 8 ± 1 | 12 | 12 |
| Diacylglycerol (DG) | 21 ± 7 | 17 | 10 |
| Triacylglycerol (TG) | 68 ± 15 | 80 | 63 |
| *Total* | ***405* ± 22** | ***486*** | ***346*** |
